# Management of Childhood Iron Deficiency Anemia in a Developed Country—A Multi-Center Experience from Croatia

**DOI:** 10.3390/diagnostics13243607

**Published:** 2023-12-05

**Authors:** Izabela Kranjčec, Nuša Matijašić Stjepović, Domagoj Buljan, Lucija Ružman, Karolina Malić Tudor, Marijana Jović Arambašić, Maja Pavlović, Nada Rajačić, Kristina Lovrinović Grozdanić, Tomislava Brković, Krešimir Šantić, Jelena Roganović

**Affiliations:** 1 Department of Oncology and Hematology, Children’s Hospital Zagreb, 10000 Zagreb, Croatia; nusa.matijasic@gmail.com (N.M.S.); domagoj.buljan23@gmail.com (D.B.); majap007@gmail.com (M.P.); nada.rajacic@kdb.hr (N.R.); 2 Department of Pediatrics, Division of Hematology, Oncology and Clinical Genetics, University Hospital Center Rijeka, 51000 Rijeka, Croatia; lucija.ruzman@gmail.com (L.R.); kristinalovrinovic87@gmail.com (K.L.G.); roganovic.kbcri@gmail.com (J.R.); 3 Department of Pediatrics, University Hospital of Split, 21000 Split, Croatia; malictudor.k@gmail.com (K.M.T.); tomislava_jukic@yahoo.com (T.B.); 4 Department of Pediatrics, University Hospital Osijek, 31000 Osijek, Croatia; marijana.arambasic@kbco.hr (M.J.A.); kresimir.santic@kbco.hr (K.Š.)

**Keywords:** iron, iron deficiency, anemia, child, infant, hematology

## Abstract

Iron deficiency anemia (IDA) continues to be a global public health concern, mostly in the developing countries. However, precise epidemiological data on childhood IDA in Croatia are lacking. In order to establish its frequency, underlying etiologies, the rationale for tertiary care visits, diagnostic practices, and current treatment regimens of IDA, medical records of children referred to pediatric hematologists for iron deficiency in a five-year period at tertiary institutions (Zagreb, Rijeka, Split, Osijek) throughout Croatia were retrospectively analyzed. Eight hundred and sixty-four children, predominately of preschool age, were referred mainly by the primary care pediatricians, who, in general, performed basic diagnostics but failed to initiate oral iron therapy in half of the patients. Approximately one-third of patients were symptomatic, with inadequate nutrition prevailing as underlying etiology. Dextriferron was the preferred iron formulation among hematologists, with a median dose of 5 mg/kg, with acceptable compliance rates (63.5–93.2%). Hospital admission rates varied among the centers (9.4–35%), and so did transfusion policies (6.4–22.9%). The greatest difference was observed in the frequency of parenteral iron administration (0.3–21.5%). In conclusion, the burden of childhood IDA, even in a high-income country, remains substantial, necessitating consistent implementation of national guidelines and additional education of primary health care providers.

## 1. Introduction

Iron deficiency anemia (IDA) continues to be a global public health concern, primarily affecting young children, female adolescents, and pregnant and postpartum women. According to the latest World Health Organization (WHO) data, approximately 40% of children aged 6–59 months and 30% of women of reproductive age are anemic, worldwide. The prevalence of anemia in youngsters under 5 years of age is the highest in Africa, at 60.2%, as iron remains the number one deficient nutrient in the developing world [1]. The lack of other microelements, chronic inflammation, parasitic infections, and inherited red blood cell (RBC) disorders additionally contribute to the magnitude of anemia [2].

Due to preventive measures, mainly the introduction of iron-fortified infant formulas and foods, IDA prevalence rates in developed countries have significantly declined over the past decades [3,4,5]. In the United States, about 1.1% of children 1–5 years of age suffer from IDA, with rates expectedly varying depending on ethnicity and socioeconomic status [6]. In Europe, IDA prevalence rates in infants and young children are considerably lower in affluent societies of Northern and Western regions (generally below 5%), compared to Eastern European countries (up to 50%) [7,8,9,10,11]. Prolonged exclusive breastfeeding and excessive cow’s milk consumption are the main factors negatively associated with hemoglobin (Hb) and serum ferritin (SF) concentrations [9,12,13].

Croatia lacks accurate epidemiological data on IDA in healthy children. A small cross-sectional study on infants in northwestern Croatia revealed rather dissatisfactory results comparable to Eastern Europe, with IDA prevalence up to 32.4% [14]. This severe IDA burden is presumed to mainly reflect inadequate implementation of national guidelines on screening and prevention of IDA in primary health care settings. The findings are alarming given that undetected iron deficiency (ID) may have long-term consequences for children’s growth and motor, cognitive and behavioral development.

Apart from improving nutrition by educating the patient and the family, the basic principle of IDA treatment consists of correcting the anemia and replenishing the iron stores with oral iron therapy, and additionally, monitoring response [15]. However, the current literature data on the best iron salt formulation, appropriate dosage, scheduling and duration are insufficient; therefore, treatment strategies vary [16]. Parenteral iron therapy should be considered in patients intolerant or non-adherent to oral iron, while blood transfusion is reserved for children with very severe IDA [15].

The aim of our study was to analyze epidemiological data and clinical and laboratory findings of children and adolescents with IDA referred to pediatric hematologists at tertiary institutions throughout Croatia. We further investigated different underlying etiologies, rationales for tertiary care visits, diagnostic work-up practices, and current treatment regimens for IDA in Croatian children.

## 2. Materials and Methods

**Figure 1:**
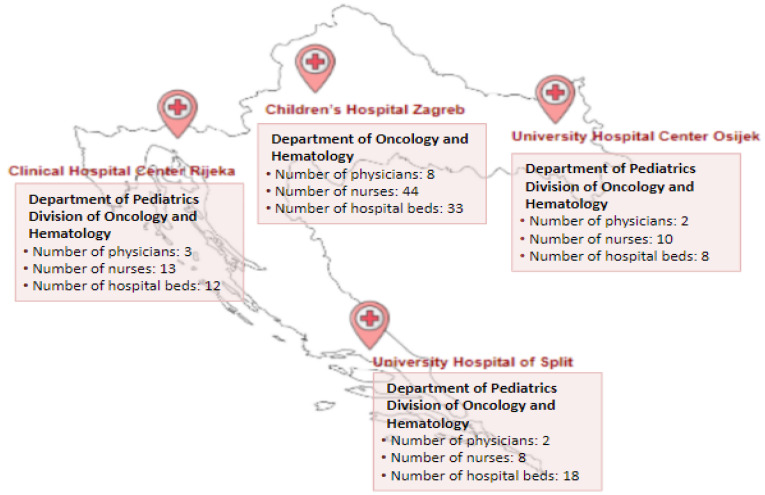
Locations and characteristics of the four Croatian pediatric hematology centers participating in the study.

A retrospective observational study was conducted in four Croatian pediatric hematology centers on patients aged 0–18 years diagnosed with iron deficiency anemia (ICD-10 D50.0–D50.9) and treated from 1 January 2017 to 31 December 2021. The geographical location and characteristics of each participating center are depicted in Figure 1.

Table 1 summarizes the World Health Organization’s (WHO) definition of anemia and iron deficiency (ID) according to age and sex.

Epidemiological (age, sex, year of the initial referral), clinical (signs and symptoms) and laboratory (hemoglobin, Hb; mean corpuscular volume, MCV; serum iron and ferritin, SF concentrations) data were retrospectively gathered from electronic medical records. Analyzed laboratory results were those obtained through testing at three different time points: initial—at the primary care level, and at the first and last tertiary care visit. Patients whose data were lacking and/or did not meet WHO diagnostic criteria for IDA were excluded from the study. Performed diagnostics (basic laboratory testing including complete blood count and iron status; iron, ferritin, unsaturated iron binding capacity /total iron binding capacity) or extended work-up (e.g., anti-tissue transglutaminase, fecal occult blood, fecal calprotectin, etc.) and therapeutic actions (introduction of oral iron, product type and dosage), taken prior to pediatric hematologist’s consultation, as well as the information on referring physician (primary care pediatrician, general practitioner, (GP), other specialists), were registered. If known, underlying causes of low iron status and consequent anemia were recorded. The association between the occurrence of symptoms and IDA etiology was examined. Table 2 summarizes the etiology of ID/IDA in children.

Information regarding the extent of diagnostic work-up performed at the tertiary care level was obtained. Data concerning therapy prescribed by pediatric hematologists included information on the type of oral iron preparation, dosage, therapy duration, patients’ compliance, and if conducted, parenteral iron therapy, transfusions, and hospitalizations.

Descriptive statistics were used to describe participant data. The equality of proportions was examined using the chi-square test. In cases where the chi-square test was not applicable, the Fisher’s exact test was used. The chi-square test was applied when examining the equality of proportions for variables with more than two categories. To determine whether differences existed, a series of post hoc tests were carried out. Statistically significant results were considered those with *p*-values under 0.05. Statistical analysis was carried out using R 4.1.1. (R Core Team, Vienna, Austria). 

The study was conducted in accordance with the Declaration of Helsinki and approved by the Institutional Review Board.

## 3. Results

### 3.1. Children’s Hospital Zagreb

Two hundred and ninety-nine patients met the inclusion criteria in the Zagreb center, with the male–to-female ratio 1:1.1. The median age was 5 years, and the two mainly affected age groups were preschoolers (94, 31.4%) and early adolescents (69, 23.1%). Table 3 shows patients’ epidemiological data for all four centers.

**Figure 2:**
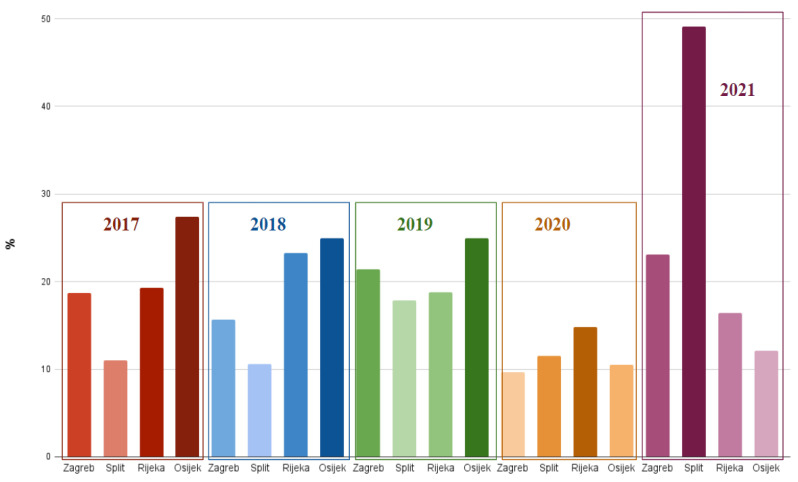
Percentage of patients referred to pediatric hematologist’s consultation annually for all four centers.

The greatest number of patients (69, 23.1%) were referred to pediatric hematologist’s consultation during the year 2021, mostly by primary care pediatricians, who were the most common referral source throughout the study period. Figure 2 displays the annual percentage of patients referred to the hematologist’s office for Zagreb and the other three hematological centers.

Results of basic laboratory tests performed before tertiary care visits and at the tertiary institution are shown in Table 4. Additional work-up, recommended by pediatric hematologists, was carried out in 63.7% of participants.

**Figure 3:**
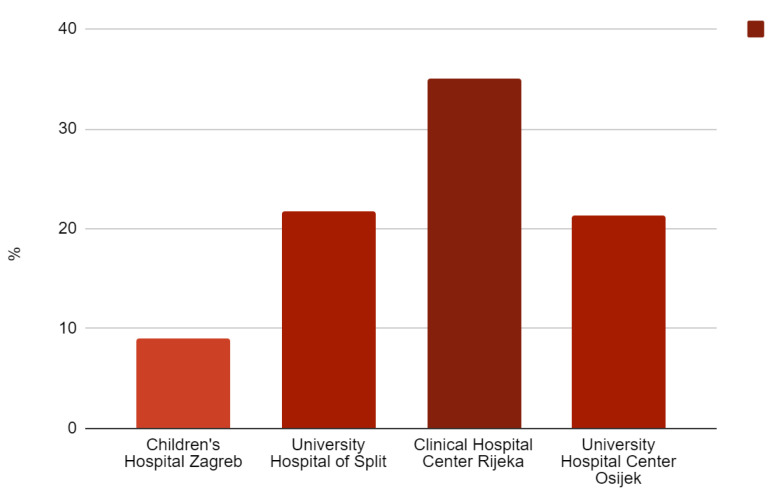
Hospital admission rates, % (all four centers included).

The most common underlying etiology was menorrhagia (67, 22.5%). Symptoms were present in 32.4% of patients (*N* = 96) and occurred more frequently in females with profound menstrual bleeding leading to IDA (*p* = 0.001). Oral iron therapy was introduced to approximately half of the patients (167, 56.6%) prior to the hematologist’s visit, after which all of the participants were adequately treated. Although other specialists were more likely to introduce oral iron (*p* = 0.019), there was no statistically significant difference in prescribing iron at the primary care level between pediatricians and GPs. Hematologists most often recommended dextriferron, with a median dose of 5 mg/kg, over a mean period of 5.8 months (median 4, range 1–27 months). Poor compliance was present in 36.5% of patients (*N* = 109). The hematologist’s follow-up typically lasted for a year and included four visits per patient on average. The hospital admission rate at Children’s Hospital Zagreb was 9.4% (*N* = 28), while only one patient was given intravenous iron therapy. Statistical analysis on severe cases of IDA requiring hospitalization and/or transfusions or parenteral iron could not be performed, given the small number of these patients. Comprising data for all four centers, Figure 3 depicts hospital admissions due to severe IDA.

### 3.2. University Hospital of Split

**Figure 4:**
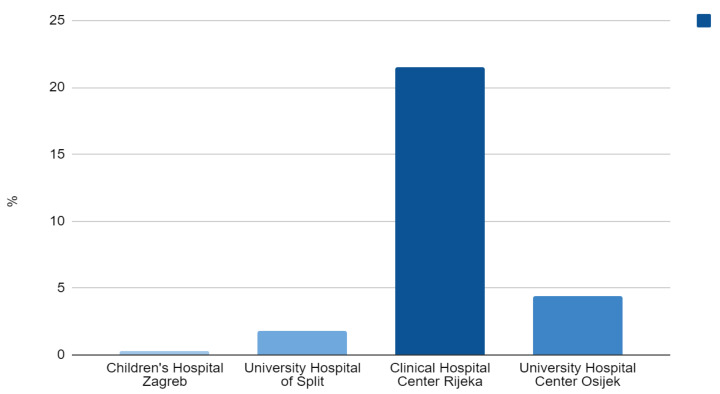
Parenteral iron therapy, % (all four centers included).

Out of 218 participants from the Split center, the majority were male (116, 53.2%), predominantly infants (111, 50.9%), followed by preschoolers (37, 17%), with a median age of 1 year. The largest percentage of patients (107, 49.1%) seen by hematologists were referred in the year 2021, mostly by primary care pediatricians who sent 69.7% of patients (*N* = 152) altogether to the clinic throughout the study’s duration. Figure 2 displays the annual percentage of patients referred to the hematologist’s office for Split and the other three hematological centers. At the tertiary care level, 22.9% of children received extended work-up (*N* = 48). Malnutrition was the most common cause of IDA, in 62% of the patients (*N* = 132). Approximately one-third of the patients (72, 35.8%) were symptomatic, significantly more often those with inadequate diet (*p* < 0.001). Less than half of the patients (74, 49%) received oral iron before being referred to a tertiary institution. Primary care pediatricians and other subspecialists prescribed iron therapy significantly more often than GPs (*p* < 0.01). The most frequently prescribed formulation was iron protein succinylate (77, 39.9%) during a mean time of 4.4 months (median 4, range 0–18 months), and the majority of patients (178, 85.6%) were compliant. The hematologist’s follow-up included four visits per patient on average. Approximately 20% of children seen by hematologists for IDA were admitted to the hospital (*N* = 47). In addition, about one-fifth of all patients received blood transfusion (*N* = 49), while parenteral iron was given to only four patients. Figure 4 shows rates of conducted parenteral iron therapy for all of the participating centers.

### 3.3. Clinical Hospital Center Rijeka

**Figure 5:**
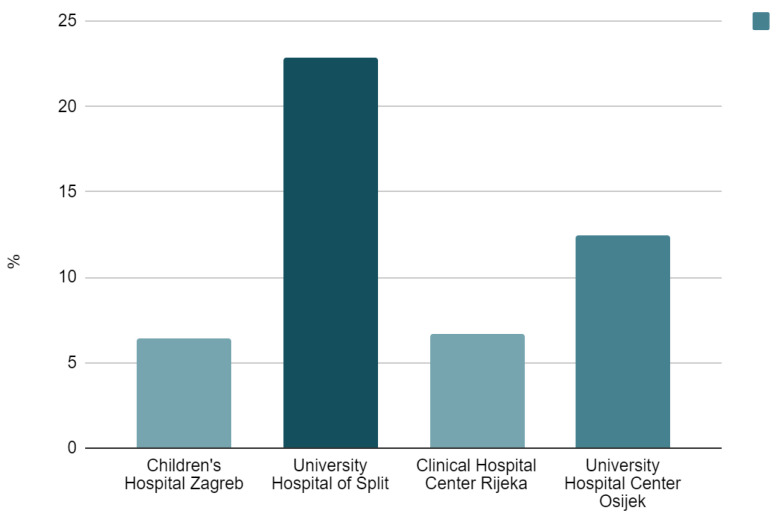
Transfusion rates, % (all four centers included).

Two hundred and twenty-three patients were treated in the Rijeka center, predominantly male (128, 57.4%), with a median age of 2.2 years. Preschool children (102, 45.7%) and infants (50, 22.4%) were mostly affected. The majority of participants were referred to the clinic by their primary care pediatrician (58.3%, *N* = 130), and 2018 was the year with the greatest number of referrals (52, 23.3%). Figure 2 displays the annual percentage of patients referred to the hematologist’s office for Rijeka and the other three hematological centers. Extended hematologist’s work-up was carried out in 48.4% of children (*N* = 108). In 38.6% (*N* = 86) of patients, IDA resulted from poor nutrition. Thirty percent of patients (*N* = 63) had symptoms, which occurred noticeably more often when the cause of IDA was menorrhagia (*p* < 0.001). In approximately 60% (*N* = 135) of patients, iron supplementation therapy started prior to the hematologist’s consultation. Primary care pediatricians prescribed iron statistically more often than GPs (*p* = 0.009), while, on the other hand, GPs introduced iron more frequently than other subspecialists (*p* = 0.005). The preferred prescribed preparation was dextriferron (114, 62%), with a median dose of 5 mg/kg, during a mean period of 3.5 months (median 3, range 0–24 months). Iron treatment was taken as prescribed by 67.7% of patients (*N* = 151). The hematologist’s follow-up included four visits per patient on average. Thirty-five percent of patients (*N* = 78) were admitted to the hospital, with no statistically significant difference regarding etiology of IDA among them. Fifteen patients (6.7%) received transfusions; in 21.5% of patients (*N* = 48), parenteral iron therapy was conducted. Patients with menorrhagia were more likely to receive intravenous iron (*p* < 0.001). Figure 5 shows transfusion rates for all four centers.

### 3.4. University Hospital Center Osijek

There were 124 participants included in the Osijek center. Patients were predominantly male (66, 53.2%), principally preschoolers (63, 50.8%), followed by adolescents (20, 16.1%), with a median age of 5 years. The majority of patients (34, 27.4%) were referred to the hematologist in 2017, mostly by primary care physicians (119, 96%) who were not, at the time of collecting data, divided into GPs and primary care pediatricians, so no comparison could be made regarding these two variables. Figure 2 displays the annual percentage of patients referred to the hematologist’s office for Osijek and the other three hematological centers. Approximately half of the patients (62, 53%) received a more extended hematological work-up at the tertiary care level. Inadequate nutrition was the leading underlying cause of IDA, detected in 45.1% of patients (*N* = 55). Symptoms were present in 38.5% of patients (*N* = 47), significantly more often when inadequate diet (*p* < 0.001) and prolonged menstrual bleeding (*p* = 0.009) or gastrointestinal disease (*p* = 0.03) caused low iron. Before being seen by hematologists, 66.7% of participants (*N* = 80) were already given oral iron, most commonly dextriferron (48, 55.2%) at an average dose of 5 mg/kg, during a mean period of 9.9 months (median 5.5, range 0–60 months) and with compliance of 93.2% (*N* = 96). The hematologist’s follow-up included two visits per patient on average. Transfusion was given to 15 (12.5%) patients, while 5 (4.4%) patients received intravenous iron. There was no statistically significant difference in etiology of IDA for patients receiving parenteral iron and/or transfusion therapy.

### 3.5. Summary Results

The total number of participants in four hematology centers was 864, with slight male predominance (*N* = 453, 52.4%). The greatest number of patients belonged to the preschool age group (*N* = 296, 34.3%), followed by infants (*N* = 224, 26%) and early adolescents (*N* = 154, 17.8%), with a median age of 3.5 years. Most referrals occurred in the second pandemic year, 2021 (*N* = 227, 26.3%), and primary care pediatricians were the major referral source (total analyzed *N* = 727; *N* = 411, 56%).

Only half of the patients (*N* = 498, 54.1%) started oral iron therapy in the primary care setting, but all were treated by the hematologist, preferably with dextriferron (total analyzed *N* = 715; *N* = 356, 49.8%) and high a compliance rate (*N* = 615; 71.2%). In almost half of the patients, broader work-up was performed in the tertiary health care setting (*N* = 402, 46.5%), unlike a small number of patients being extensively laboratory-processed at the primary level (*N* = 64, 7.4%). One-third of all patients were symptomatic (total analyzed *N* = 842; *N* = 278, 33%), and in slightly more than one-third, poor nutrition was recognized as the etiology of IDA (total analyzed *N* = 824; *N* = 318, 38.6%).

Every fifth child was admitted to the hospital (*N* = 179, 20.7%), and every tenth received a blood transfusion (*N* = 98, 11.3%), while intravenous iron therapy was seldom administered (*N* = 58, 6.4%).

## 4. Discussion

According to the WHO estimate from the beginning of the century, about one-quarter of Croatian preschool children and pregnant women suffered from IDA [17]. However, precise data on the prevalence of IDA in pediatric and adult female populations in Croatia are still deficient, suggesting that IDA has not been recognized as a potentially severe public health concern on a national level.

Notwithstanding the paucity of methodical studies over the last two decades, a recent observational survey in a primary health care setting in central Croatia reports IDA prevalence of 12% in children above 1 year and around 18% in early pregnancy in the Eastern part of the country [18,19]. A declining trend most likely reflects a rising standard of living since the association of IDA and socioeconomic vulnerability has long been established worldwide [20,21,22,23].

Although IDA prevalence in children in Croatia nowadays most likely has gradually approached low rates in other developed European countries [23], the burden of childhood IDA cases provided for in the tertiary institution remains high. Namely, the results of our survey show that more than eight hundred pediatric patients were seen by the hematologist in a five-year period in four centers throughout the country. Most referrals in the capital and second-largest city in Croatia, Split, occurred in the second pandemic year when hospital care was once again readily available for non-urgent, COVID-unrelated medical conditions. In the remaining two centers, the influx of pediatric IDA cases to the tertiary institutions was up most years before the pandemic, probably reflecting the current healthcare system structure, number of personnel and workload.

As persistent, unexplained microcytic anemia or anemia refractory to iron supplementation is generally considered a valid indication for hematologist and/or gastroenterologist referral [24], the fact that in approximately only half of the patients, oral iron therapy was initiated prior to referral is quite concerning. According to the results of the small survey among primary care physicians during an educational session on benign hematological disorders in children, IDA is the condition the colleagues encounter most frequently and feel most secure managing in everyday practice, so the high number of diagnosed patients left untreated is both puzzling and alarming [25].

Recommendations on IDA screening, diagnostics and therapy were provided by the American Academy of Pediatrics (AAP) more than ten years ago; moreover, the negative impact of IDA on neurodevelopment in infants and older children has consistently been observed over the decades [5,26,27]. Free medical health care for children and adolescents and easily accessible tertiary health services with acceptable waiting lists might be one of the reasons for the high referral rate. Whether knowledge on anemia management gained during residency for primary pediatricians, and in particular, during specialty training for family medicine doctors, is sufficient might be a matter of debate since other hospital specialists (e.g., gynecologists) were significantly more alert in prescribing upfront iron therapy. Furthermore, a lack of national recommendations on childhood IDA management by the pediatric society most certainly contributes to primary health care providers’ insecurity. Joined Croatian Hematology Society’s and Croatian Cooperative Group for Hematologic Diseases’ (CroHem’s) guidelines from 2019 are oriented primarily towards adult IDA diagnostics and treatment and appear to be well-known only to a narrow circle of hematologists [26]. Nevertheless, even in developed countries with abundant IDA treatment recommendations by various committees and physicians’ associations, such as the United States, the number of patients seeking medical advice in hematology clinics is surprisingly high, and the proportion of children with instantly prescribed iron therapy is below 50%, highlighting the necessity for prospective, controlled studies in all age groups and the establishment of evidence-based treatment practices [28].

Universal screening for IDA is recommended by the AAP in infants at the age of 12 months, and includes Hb measurements with risk factor assessment (low socioeconomic status, inadequate nutrition, feeding problems, exclusive breastfeeding, prematurity, low birth weight, etc.) [5]. Additional screening for toddlers is advised anytime until the third year of life [5]. CroHem’s guidelines are similarly in favor of routine screening in all children between the 6th and 24th month of age, but according to the small-scale survey in the Croatian capital’s surroundings, it is implemented in everyday practice in less than half of infants provided for in the primary pediatrics office [29]. However, the United States Preventive Services Task Force and the United Kingdom National Screening Committee, for example, do not support universal screening of infants and preschool children below 5 years of age, nor does the Swiss Pediatric Hematology Working Group, but only in the presence of signs and symptoms of anemia or ID [26,30]. Moreover, in most of the recommendations, the focus on IDA screening has been put on infants and young toddlers, primary risk groups, while other childhood age clusters have been somewhat neglected, hence the possible explanation for the higher median age in our total sample and three out of four investigated centers. If a decreased Hb level has been established at screening, according to the AAP, SF with C-reactive protein (CRP) should be determined [5]. Nevertheless, neither SF nor extensive work-up regarding etiology of anemia can be performed in the laboratories affiliated with primary health care centers in Croatia, which might be an additional reason for the high referral rate.

There was a significantly higher tendency for hospital admissions in Rijeka compared to Zagreb, although the proportion of symptomatic cases was the same, approximately one-third of the patients. However, even the highest hospitalization rate in Rijeka is still below 50% reported in the adult population in the USA and remarkably above 2.6%, 4% and 3.9% in Brazilian infants, children under 10 years and adolescents, respectively [31,32]. Since prolonged or profuse menstrual bleeding was the prevailing underlying etiology among patients in Zagreb, and inadequate nutrition in remaining centers, the highest number of children treated in the in-hospital setting would be expected in the capital, yet that was not the case. This discrepancy cannot be explained by health care resource statistics either, but most likely reflects the physicians’ personal clinical experience and IDA management approach, which are related to the higher rate of parenteral iron therapy. Our data on admission rate trends in five years are not unavailable; however, a recent study from the United States informs of a worrisome growing hospitalization course [33].

A considerable number of oral iron drugs are available worldwide, but ferrous sulfate is generally considered a standard of treatment [34,35]. Iron salts in ferrous (+2) forms have better absorption than ferric (+3) preparations, and trials comparing different oral formulations regarding Hb and SF increase rate favor in particular ferrous sulfate [36,37]. Bis-glycinate iron preparation might present an efficient, safe, and acceptable alternative to ferrous salts [35]. However, most of our patients were treated with a ferric salt, dextriferron, since it is the only medicine among three registered in Croatia (ferrous hydroxide, ferrous sulfate, ferrous glycine sulfate) that is covered by the national insurance company [38]. Due to adverse events, mainly affecting the gastrointestinal tract, a substantial number of adolescent patients and their parents in the Zagreb center self-initiated therapy modification, shifting to iron formulations that are registered as dietary supplements, containing negligible amounts of iron (0.75–1.7 mg/mL), most likely contributing to therapy prolongation, and failure. Adherence to oral iron therapy ranged from approximately 60 to 90%, depending on the center, and is consistent with or slightly above the rate reported in the literature [28,39]. Barriers to satisfactory compliance most commonly reported in the literature are mainly poor taste and side effects of oral preparations [40], but these were not thoroughly investigated in our cohort. Because optimal iron dosing regimens in children have not been studied in detail in controlled, randomized trials, the dosing recommendations vary; most textbooks and papers suggest 3 mg Fe/kg body weight in younger children and 65–130 mg in adolescents [34,41,42,43]. The Swiss Pediatric Hematology Working Group distinguishes dosing regimen regarding Fe state, so 2–3 mg/kg of ferrous and 3–5 mg/kg of ferric elemental iron supplementation are suggested [26]. Nevertheless, hematologists in the United States tend to prescribe higher amounts of daily iron, reaching up to 6 mg/kg in two daily doses in younger children, as was the case with Croatian colleagues, who most likely complied with CroHem’s guidelines [28,44,45]. Iron preparations should be ingested between meals, and avoiding uptake inhibitors is firmly recommended [46]. Once-daily dosing is advised by some experts in the field to minimize gastrointestinal intolerance, promote iron absorption and improve therapy adherence [34]. In order to further improve iron bioavailability, complementing therapy with vitamin C was previously suggested, however, without sufficient evidence while aggravating digestive side-effects [46,47]. Recently, the addition of lactoferrin showed promising results in replenishing iron stores in children [48].

In women with heavy menstrual bleeding, intravenous iron as first-line therapy is recommended in cases of heavy anemia or those necessitating prompt improvement, e.g., before urgent surgery, and as a second-line therapy in cases of poor adherence, intolerability or deficient therapy response [49]. Unsuccessful oral iron therapy in children is likewise considered a justified indication for parenteral therapy, and in certain groups of patients, for example, children with chronic kidney and gastrointestinal disease and those on extended parenteral nutrition, intravenous iron formulations provide an appropriate upfront therapeutic alternative [50]. Menorrhagia was the principal reason for parenteral iron therapy in the Rijeka center, where most of the intravenous iron medications were administered. Tremendous differences in parenteral iron therapy applications among centers, especially Rijeka and Zagreb, might be due to the institutional policies and physicians’ attitudes on IDA treatment, and not only the underlying cause, degree of IDA, and response to oral iron. Moreover, some studies emphasize that parents’ fears associated with parenteral iron are obstacles that are easily overcome by deferring the decisions to the health care professional [40,41]. Safety concerns among our pediatric hematologists, especially with formerly used high molecular weight dextran, were highly likely the principal reason for the low parenteral iron application rate, as is probably the case with their American colleagues [45]. Since multiple studies report ferric carboxymaltose preparations as a safe, well-tolerated and efficacious measure to treat IDA in children who fail to respond to oral iron therapy regardless of the etiology, pediatric hematologists should not refrain from their use [51,52,53]. Nevertheless, bearing in mind the average age of our study’s participants, the underrepresentation of parenteral iron as a therapeutic option is not at all surprising given the fact that ferric carboxymaltose has only recently been approved for use in children above 1 year of age in Croatia, as in the United States [50]. Although iron sucrose has proved equally effective with an acceptable safety profile, according to the drug’s prescription instructions, its use in children is still not encouraged in Croatia [54,55]. Novel parenteral iron therapeutics are emerging for adults with IDA that might soon be available for pediatric patients. Ferumoxytol, for example, has proved to be equally efficient in ameliorating anemia and improving iron stores such as iron sucrose and ferric carboxymaltose [56]. Nevertheless, as with the oral iron therapy recommendations, large prospective, randomized trials in the pediatric setting are yet to be performed.

The decision to transfuse adolescent girls with IDA due to metrorrhagia is not based solely on Hb levels but on the presence of symptoms and hemodynamic instability [49]. Transfusion in hemodynamically stable children with IDA related to other etiologies is also generally discouraged by the American Association of Family Physicians, American Society of Hematology and American Society of Pediatric Hematology/Oncology [57,58]. Despite the significant discrepancies between Zagreb and Rijeka centers in the rate of parenteral iron use, the two centers were equally reluctant regarding packed RBC administration, in contrast to the center in Split, which reported a rather high percentage of transfused patients, similarly to its Canadian counterparts [59]. Although straightforward clinical guidelines were published by the aforementioned hematology societies only recently, suggestions to avoid transfusion therapy in children with IDA whenever feasible have been available on the websites of many distinguished pediatric medical health centers in North America [60,61,62]. Data on transfusion therapy rates over the observed period in the Split center are, unfortunately, not available, but a declining trend would be expected according to the reports from the literature [40,63].

More than a third of patients were lost to follow-up, although IDA had not resolved at the time of their last visit according to the results from a Texas study [28]. Our study’s average number of visits was three per patient (the maximum number ranging from nine in Osijek to 23 in Zagreb), with a median SF level at the time of the last follow-up slightly or moderately above the diagnostic threshold. However, a significant proportion of children left the hematologists’ office with SF levels of 3 µg/L; thus, a risk for potential neurodevelopmental disorders after final consumption of health care services has not been entirely avoided. Namely, the negative impact of IDA on psychomotor development and cognitive function has been long recognized [64,65]. Recent studies report that gross motor and adaptability development is negatively correlated with IDA in infants and toddlers, especially attention deficit hyperactivity disorder (ADHD) and autism spectrum disorder (ASD) [66,67]. A higher susceptibility to febrile seizures in children with ID and IDA has also been determined in a large number of case–control studies [68]. Not only does IDA adversely affect development and growth, but it might also contribute to hypercoagulability due to oxidative stress, endangering young patients with thromboembolic events [69,70].

We are aware of the possible limitations of our study. Firstly, the retrospective nature provides an inferior level of evidence. Medical records were often incomplete, preventing any elaborate statistical analysis of variables of interest. Secondly, for the same reason, no distinction was made between ID and IDA; our results solely reflected anemia characteristics, diagnostics and management.

Nonetheless, this is the first study to investigate the burden of childhood IDA in Croatia. Elaborate data on a substantial number of participants in geographically diverse parts of the country offer a detailed insight into IDA management practices in four tertiary pediatric hematology centers in a developed, south European country.

## 5. Conclusions

Many children and adolescents are still referred to a hematologist due to IDA. Oral iron supplementation fails to be prescribed by the primary care physician in a significant proportion of patients prior to referral. Most of our participants were treated with ferric formulations in higher recommended doses with satisfactory adherence rates. The percentage of children admitted to hospital for transfusions or administered parenteral iron therapy varies considerably among centers, reflecting institutional policies and physicians’ personal attitudes toward IDA.

The results of our survey support oral iron therapy as a successful first-line treatment option for childhood IDA that can be generally well-managed in the primary healthcare setting. Dietary modifications are an essential complementary tool in managing IDA, as poor nutrition continues to be a leading cause of iron deficiency in children. Hospital admission, intravenous iron administration, and blood transfusion are seldom necessary and primarily for severe IDA cases.

However, prospective, controlled studies on IDA treatment in children and adolescents are required, as well as widely available national guidelines, to optimize the diagnostics and treatment of this most common type of nutritional anemia.

## Figures and Tables

**Table 1:** WHO definition of anemia and ID.

Anemia	
Age and Sex	Definition
6–59 months	Hb < 110 g/L
5–11 years	Hb < 115 g/L
Adolescent girls	Hb < 120 g/L
Adolescent boys	Hb < 130 g/L
**Iron Deficiency**	
**Age**	**Definition**
Infants and preschool children (0–59 months)	SF < 12 mcg/L
School-age children and adolescents (5–19 years)	SF < 15 mcg/L

Abbreviations: Hb = hemoglobin; SF = serum ferritin.

**Table 2:** Etiology of ID/IDA in children.

Etiology	Example
Dietary factors	Malnutrition, formula (if iron-fortified), excessive cow milk consumption, goat milk consumption
Gastrointestinal disease	Celiac disease, inflammatory bowel diseases
Bleeding	Profound menstrual bleeding, von Willebrand disease, platelet disorders, hemophilia

**Table 3:** Epidemiological data (total number of study participants, *N* = 864).

Centers	All Four Centers	Children’s Hospital Zagreb	University Hospital of Split	Clinical Hospital Center Rijeka	University Hospital Center Osijek
Sex					
Male (*N*, %)	453 (52.2)	156 (52.2)	116 (53.2)	128 (57.4)	66 (53.2)
Female (*N*, %)	411 (47.8)	143 (47.8)	102 (46.8)	95 (42.6)	58 (46.8)
*p*-value	0.15	0.45	0.34	0.03	0.47
Age					
Mean	6.5	7.5	5.0	5.6	7.7
Median	3.9	5.0	1.0	2.2	5.0
0–12 months (*N*, %)	224 (26)	56 (18.7)	111 (50.9)	50 (22.4)	7 (5.7)
1–4 years (*N*, %)	296 (34.3)	94 (31.4)	37 (17)	102 (45.7)	63 (50.8)
5–9 years (*N*, %)	59 (6.8)	20 (6.7)	18 (8.3)	7 (3.1)	14 (11.3)
10–14 years (*N*, %)	154 (17.8)	69 (23.1)	33 (15.1)	33 (14.8)	19 (15.3)
15–18 years (*N*, %)	131 (15.1)	60 (20.1)	19 (8.7)	31 (14)	21 (16.9)
*p*-value	<0.05	<0.05	<0.05	<0.05	<0.05

**Table 4:** Results of basic laboratory work-up.

Laboratory Values		Prior to the Hematologist’s Visit			
		Zagreb	Split	Rijeka	Osijek
Hb (g/L)	Mean	97.7	97.0	85.5	103.9
	Median	96.0	97.0	86.0	106.0
MCV (fL)	Mean	71.4	72.5	61.3	62.3
	Median	71.8	73.0	58.7	66.7
Fe (μmol/L)	Mean	6.5	6.8	4.6	6.0
	Median	4.0	5.0	3.0	3.9
**Laboratory Values**		**Hematology Center**			
		**Zagreb**	**Split**	**Rijeka**	**Osijek**
Hb (g/L)	Mean	105.0	102.5	94.5	109.0
	Median	106.0	103.6	96.0	108.5
MCV (fL)	Mean	73.0	74.0	66.1	65.4
	Median	74.5	74.2	65.7	70.0
SF (μmo/L), initial	Mean	15.1	34.1	13.2	21.6
	Median	7.0	9.0	10.0	12.1
SF (μmo/L), final	Mean	21.2	28.7	38.5	34.9
	Median	17.7	22.0	23.5	21.2

Abbreviations: Fe = iron; Hb = hemoglobin; MCV = mean corpuscular volume; SF = serum ferritin.

## Data Availability

The data presented in this study are available on request from the corresponding author. The data are not publicly available due to patient privacy and confidentiality policy.
